# Supplementary material for: Diagnostic Accuracy of Ischemia-Modified Albumin for Acute Coronary Syndrome: A Systematic Review and Meta-Analysis
Source: Medicina (Kaunas). 2022 Apr 28;58(5):614. doi: 10.3390/medicina58050614 (PMC9143213; doi:10.3390/medicina58050614)

Table S1. Patients characteristics and definition of ACS in included studies.

Author	Year	Diagnosis	N	Criteria
Aggarval	2012	Total	100	● After obtaining informed consent from the patients and approval from the institutional board of studies, the present study was conducted on 100 patients of acute chest pain (age group 30-60 years) visiting the Emergency Department of Pt.B.D.Sharma PGIMS, Rohtak within 6 hours of chest pain and 50 healthy controls.<br>● Patients having renal disease, liver disease, pregnancy, brain ischemia and trauma were excluded from the study.<br>● These patients were divided into three groups based on electrocardiography (ECG) changes and clinical signs and symptoms.
		NICP	50	-
		ACS	50	-
Anwaruddin	2005	Total	193	● Data were collected for 200 consecutive patients admitted to an urban ED with manifestations suggestive of acute myocardial ischemia, including those such as chest pain with or without radiation, chest pressure, shortness of breath, lower jaw pain, left arm pain, epigastric pain, syncope, hypotension, new or increasing lower extremity edema, palpitations, and other symptoms suggestive of an anginal equivalent.
		NICP	168	● -
		ACS	25	● Ischemic syndromes included unstable angina, non–ST segment elevation myocardial infarction (MI), and ST segment elevation MI. The definition of MI was as previously described.<br>[Alpert JS, et al. Myocardial infarction redefined—a consensus document of The Joint European Society of Cardiology/American College of Cardiology Committee for the redefinition of myocardial infarction. J Am Coll Cardiol. 2000;36:959-69.]
Bhakthavatsala	2014	Total	89	● Data were collected from this 89 patients admitted to our emergency department with manifestations suggestive of acute myocardial ischemia, including those such as chest pain with or without radiation, chest heaviness, shortness of breath, lower jaw pain, left arm pain, epigastric pain, syncope, hypotension, palpitations, and other symptoms suggestive of an anginal equivalent.<br>● Inclusion criteria<br>(1) Patients admitted in the emergency room with a primary complaint of chest pain or angina equivalence evolving within 6 h and suspected as acute coronary syndrome.<br><br>● Exclusion criteria<br>(1) Presence of renal diseases.<br>(2) Presence of cirrhosis.<br>(3) Presence of stroke, skeletal muscle injury, malignancy, trauma.<br>(4) Ongoing infectious diseases.<br>(5) Serum albumin <2 g/dl.<br>(6) Patient younger than 18 years old.<br>(7) Patients with complaints lasting more than 6 h, as IMA levels usually return to normal 8-12 h after onset of

				myocardial ischemia.<br>(8) Patients whose symptoms had ceased 2 h previously, because IMA levels fall rapidly once an ischemic event has ended.<br>(9) Asymptomatic patients, and those who unable to relate the time that their symptoms began or ended (if the pain was not persisting).
		NICP	24	● Patients were classified as nonischemic chest pain (NICP) when<br>(1) a reported non-cardiac mechanism was confirmed as the cause of chest pain;<br>(2) all of the following criteria were met: atypical symptoms, negative cTnT results on serial sampling (over a 6-9 h interval), presence of normal ECGs, and negative stress test.
		ACS	65	● Practice guidelines for the redefinition of AMI (ESC/ACC) and the management of patients with UA (ACC/AHA), were used to diagnose ACS.<br>[The Joint European Society of Cardiology and American College of Cardiology Committee. Myocardial infarction redefined e a consensus document of the Joint European Society of Cardiology/American College of Cardiology committee for the redefinition of myocardial infarction. J Am Coll Cardiol. 2000;36:959-69.]<br>[Braunwald E, et al. ACC/AHA guidelines for the management of patients with unstable angina and non-ST segment elevation myocardial infarction. J Am Coll Cardiol. 2000;36:970-1062.]<br>● STEMI was diagnosed if there was ST segment elevation [greater than or equal to] 0.1 mV in two or more contiguous leads.<br>● NSTEMI was diagnosed if ECG was non-diagnostic and cTnT positive.<br>● UA was diagnosed in the presence of signs and symptoms of acute cardiac ischemia without evidence of myocardial necrosis. Positive indications for UA were a suggestive history and clinical examination; typical ischemic ECG changes at rest or during exercise; regional wall motion abnormality on echocardiography, significant stenosis (>70%) on coronary angiography.
Bhardwaj	2011	Total	318	● Those consenting patients presenting with symptoms suggestive of a possible ACS meeting inclusion and exclusion criteria were enrolled.
		NICP	256	-
		ACS	62	● The diagnosis of ACS was judged using standard criteria; in brief, an ACS was diagnosed when a patient had typical angina at rest, a deterioration of previously stable angina, a positive result on a cardiac exercise test, or cardiac catheterization showing coronary arteries with stenosis of ≥70% of vessel diameter.<br>● An “acute MI” was diagnosed in the context of an ACS when there was evidence of myocardial necrosis, with a rising or falling pattern of the local conventional troponin T (cTnT) level, with at least 1 value >0.03 ng/mL.
Charpentier	2010	Total	677	● This prospective multidisciplinary study was carried out from May 2006 to March 2007 in the ED of our University Hospital in collaboration with the cardiology and clinical

				chemistry departments.<br>● Patients admitted to the Toulouse University Hospital ED within 12 hours of the last episode of chest pain, and who were suspected of having ACS by the EP, were included.<br>● Exclusion criteria were age less than 18 years, ST elevation on a 12-lead electrocardiogram (ECG), evident traumatic cause of chest pain, skeletal muscle injury within 7 days, previous severe renal impairment, or severe communication problems making it difficult to obtain informed consent. Patients without chest pain and with dyspnea or anginal equivalents were also not included in this study.
		NICP	492	● -
		ACS	185	● Patients were considered to have ACS when a diagnosis of non-ST-elevation myocardial infarction (NSTEMI) or UA was confirmed, based on current international guidelines.<br>[Bassand JP, et al. Guidelines for the diagnosis and treatment of non-ST-segment elevation acute coronary syndromes. Eur Heart J. 2007; 28:1598–660.]<br>[Thygesen K, et al. Universal definition of myocardial infarction. J Am Coll Cardiol. 2007; 50:2173–95.]<br>[Anderson JL, et al. ACC/AHA 2007 guidelines for the management of patients with unstable angina / non-ST-Elevation myocardial infarction: a report of the American College of Cardiology / American Heart Association Task Force on Practice Guidelines. J Am Coll Cardiol. 2007; 50:e1–e157.]<br>● NSTEMI was diagnosed by the presence of symptoms of myocardial ischemia and an elevated level of troponin on serial testing according to the universal definition of MI. An increased value for cardiac troponin was defined as exceeding 0.1 ug / L, the 99th percentile of a normal reference population for our clinical laboratory.<br>● UA was diagnosed based on any one of the following criteria: suggestive history and clinical examination, dynamic ischemic ECG changes at presentation or during exercise, regional wall motion abnormality following stress echocardiography, positive radionuclide scan; stenosis greater than 70% on coronary angiography, admission for MI within 30 days, or death with no other definite cause during index hospitalization or within 30 days.
Christenson	2001	Total	224	● All patients arrived at the Emergency Department at participating centers within 3 h of clinical signs and symptoms of acute coronary syndrome, as determined by medical record review.
		NICP	189	● Cardiac troponin negative patients
		AMI	35	● Cardiac troponin positive patients
Collinson	2006	Total	538	● We conducted a prospective observational study of consecutive admissions to the ED with undifferentiated chest pain.<br>● Exclusion criteria for the protocol were:<br>(1) significant ECG changes (unless known to be old): ST segment elevation >1 mm or T wave inversion in two

				continuous leads; atrial fibrillation; tachydysrhythmia >120 beats/min; bradycardia <40 beats/min; second or third degree heart block or left bundle branch block<br>(2) comorbidity requiring hospital admission suspected or proved alternative source of chest pain requiring hospital admission<br>(3) known coronary artery disease (CAD) with unstable angina<br>(4) atypical clearly non-cardiac chest pain (stabbing, pleuritic, positional, or reproduced by palpation) in a patient with no history of and few risk factors for CAD.
		NICP	501	-
		AMI	37	● To allow comparison with current recommendations, we used the European Society of Cardiology/American College of Cardiology (ESC/ACC) criteria to diagnose AMI, with cTnT as the diagnostic cardiac biomarker (cut-off of >0.05 mg/l, the optimised 10% coefficient of variation (CV) cut-off from a large multicentre study) in the admission, 6 hour, or 72 hour samples as appropriate.<br>[Collinson PO, et al. Multicentre evaluation of the diagnostic value of cardiac troponin T, CK-MB mass, and myoglobin for assessing patients with suspected acute coronary syndromes in routine clinical practice. Heart. 2003;89:280–6.]
Gurumurthy	2014	Total	540	● The patients were enrolled in the study on arrival at the ED with chest pain.<br>● The study group included 675 subjects of whom 540 were patients admitted with chest pain with or without radiation, palpitations, shortness of breath, lower jaw pain, left arm pain, epigastric pain, hypotension, and other symptoms suggestive of acute myocardial ischemia.<br>● Of the 540 patients admitted, there were 135 patients with ST segment elevation myocardial infarction (STEMI), 135 with non-ST segment elevation myocardial infarction (NSTEMI), and 135 with unstable angina (UA). The other 135 were noncardiac chest pain patients (NCCP).<br>● Patients with a malignant tumor, liver disorders and renal disorders, chronic inflammatory disorders, chronic rheumatoid arthritis, brain ischemia, acute mesenteric ischemia and pregnant women were excluded from the study.
		NICP	135	-
		ACS	405	● Myocardial infarction included STEMI and NSTEMI with typical chest pain persisting for at least 30 min, ST-segment elevation >0.2 mV in at least 2 contiguous leads, and increased serum troponin I (>0.3 ng/mL).<br>● UA was defined as ischemic chest pain at rest within the preceding 48 h or within the past month (Braunwald class II and III).
Hjortshoj	2010	Total	107	● The study population comprised 107 subjects who were admitted with chest pain and suspected of ACS during a 4-month period (February to May 2007).
		NICP	72	-
		ACS	35	● Patients were classified as having acute MI (AMI) according to the new universal definition of AMI.

				[Thygesen K, et al. Universal definition of myocardial infarction. Circulation. 2007;116:2634-53.]<br>● These included detection of rise and/or fall in cTnT above the 99th percentile of the upper reference limit (> 0.03 μg/L) together with signs indicative of the following:<br>● Relevant clinical symptoms of ischemia<br>● New ST-segment changes in the ECG<br>● Development of new Q waves in the ECG<br>● Echocardiographic signs of new regional motion defects.<br>● Patients were excluded, if they had a documented MI within the last week before admission. Patients who were on hemodialysis or had jaundice were also excluded from the study.
Keating	2006	Total	277	● Consecutive adult patients presenting with features of possible ischaemic cardiac chest pain were eligible at the discretion of the consenting physician. Included patients were required to have a normal electrocardiogram (ECG), which was defined as the absence of all of the following:<br>1. ST segment elevation or depression >0.5 mm.<br>2. T-wave inversion >1 mm (in leads other than III, aVR and V1).<br>3. Left bundle branch block.<br>● Exclusion criteria<br>The following patients were excluded:<br>1. Patients who had been in pain for > h on admission, because existing protocols specify immediate troponin analysis in this group.<br>2. Patients whose pain had ceased >2 h previously, because IMA levels fall rapidly once an ischaemic event has ended.<br>3. Asymptomatic patients, and those unable to relate the time that their symptoms began or ended (if the pain was not persisting).<br>4. Pregnant patients.<br>5. Patients on renal replacement therapy and those clinically diagnosed to have jaundice, as these conditions are known to influence IMA.
		NICP	235	-
		AMI	42	● The index test (measurement of IMA and troponin I (TnI) at presentation) and reference standard (delayed TnI measurement, taken at least 8 h after pain onset) were applied to all recruited patients.
Kim	2010	Total	367	● We prospectively screened 490 consecutive subjects with acute chest pain who arrived at the emergency department within 6 h of pain onset from November 2005 to August 2007.<br>● Inclusion criteria were chest pain developed within 6 h prior to admission and chest pain that matched at least one of the three features of unstable angina according to Braunwald's [19] classification:<br>(1) angina occurring at rest and lasting >20 min;<br>(2) new-onset angina of at least Canadian Cardiovascular Society classification [20] class III severity, and<br>(3) occurring with a crescendo pattern.<br>[Braunwald E, et al. Unstable angina: a classification. Circulation. 1989;80:410–4.]

				[Campeau L, et al. Grading of angina pectoris (letter). Circulation. 1976;54:522–3.]<br>● One hundred patients were excluded from analysis due to renal failure (serum creatinine >1.5 mg/dl), lung disease, peripheral artery disease, mesenteric ischemia, or a history of percutaneous coronary intervention, coronary artery bypass graft, or ischemic stroke.
		NICP	162	● Patients were classified as having NICP (referred to as the NICP group) when<br>(1) a noncardiac mechanism was identified as the cause of the chest pain;<br>(2) creatinine kinase (CK)-MB or cardiac troponin-T results were negative (CK-MB <5 ng/ml, troponin T <0.01 ng/ml) on serial sampling,<br>(3) the ECG showed no signs of ischemia (i.e. ST-segment depression and T-wave inversion),<br>(4) coronary imaging studies or stress tests showed no sign of significant coronary artery disease.
		ACS	205	● STEMI was diagnosed if there was ST-segment elevation >0.1 mV in two or more contiguous leads and cardiac troponin-T level >0.01 ng/ml.<br>● Non-STEMI was diagnosed if the level of cardiac troponin T was elevated (> 0.01 ng/ml) in the absence of ST-segment elevation on the ECG.<br>● Unstable angina was diagnosed in the presence of signs and symptoms of acute cardiac ischemia fulfilling Braunwald's classification and positive stress test (treadmill exercise test or adenosine stress 99m Tc-sestamibi scan) or significant stenosis (>70%) on coronary angiography.
Kountana	2013	Total	33	● Patients presenting to the emergency department with acute chest pain lasting <3 h suggestive of ACS, with normal or nondiagnostic ECG at presentation and serum levels of CK-MB and troponin within the normal range at presentation were enrolled in the present study.<br>● Reasons for excluding patients from the study<br>Abnormal electrocardiogram, Elevated serum creatine kinase-MB or troponin levels, Declined admission, Chronic kidney disease, Chronic liver disease, Other causes.
		NICP	28	-
		ACS	5	-
Lee	2007	Total	413	● The study subjects were 413 cases who underwent IMA test (208 males, 205 females) and were selected among the adults who visited the emergency room for symptoms suspicious ofACS such as chest pain, epigastric pain, dyspnea, syncope, and arrhythmia from June 2005 to May 2006.
		NICP	284	● Patients were divided into 5 groups according to their diagnosis: ACS group including unstable angina (UA) and acute myocardial infarction (AMI) (group 1; n=129), coronary artery diseases (CAD) excluding ACS (group 2; n=91), cardiac diseases excluding CAD (group 3; n=78), ischemia in areas other than the heart (group 4; n=20), and other diseases (group 5; n=95). Groups 2–5 were combined as the non-ACS groups.

		ACS	129	● The diagnosis was made by emergency medicine specialists and cardiologists and was based on the combination of clinical manifestation, ECG, cardiac markers, coronary angiography, and echocardiography.
Liyan	2009	Total	108	● We recruited, on a prospective sequential basis, patients who arrived at the emergency departments (ED) within 12 hr after the onset of chest pain.<br>● Pregnant women, patients with symptoms and signs suggestive of acute mesenteric ischemia, acute renal failure, peripheral vascular disease, or brain ischemia were not enrolled in the study.
		NICP	26	● Patients were classified as NICP when<br>(1) a report noncardiac mechanism was confirmed as the cause of chest pain;<br>(2) both of the following criteria were met: presence of normal ECGs, and absence of any current (lesions o70% diameter reduction in any major epicardial vessel) or previous evidence of CAD;<br>(3) a diagnosis of ACS was objectively excluded after admission to the coronary care unit.
		ACS	82	● Final diagnosis for this study was based on ED discharge diagnosis for patients discharged from the ED, and hospital discharge diagnosis, for patients admitted to the hospital for further investigation and management. The ED consultant or medical consultant was responsible for assigning a final diagnosis on the basis of history, clinical examination, and data from medical records. These included results of ECG, treadmill exercise test, and coronary angiography, as available. Results of all investigations were reviewed blind to IMA and H-FABP results.
Roy	2004	Total	131	● We assessed 131 patients with suspected ACS attending the emergency department (ED) at St. George's Hospital, London, UK, recruited prospectively and consecutively between December 2000 and June 2001.<br>● Patients who met all of the following criteria were selected for study:<br>(a) arrival to the ED within 3 h of last chest pain episode,<br>(b) a normal or non-diagnostic ECG on arrival and<br>(c) negative cardiac troponin results on ED admission.
		NICP	67	● Non-ischemic chest pain was diagnosed when<br>(1) a non-cardiac mechanism was documented as the cause of chest pain;<br>(2) all of the following criteria were met: negative cardiac troponin results on serial sampling (over a 6–12 h interval) and absence of any current evidence of coronary artery disease.
		ACS	64	● Patients were considered to have ACS when a diagnosis of non-ST segment elevation myocardial infarction or unstable angina was confirmed based on current international guidelines;<br>[Alpert JS, et al. Myocardial infarction redefined—a consensus document of The Joint European Society of Cardiology/American College of Cardiology Committee for the redefinition of myocardial infarction. J Am Coll Cardiol. 2000;36:959-69.]<br>[Braunwald E, et al. ACC/AHA guideline update for the

				management of patients with unstable angina and non-ST-segment elevation myocardial infarction—2002: summary article: a report of the American College of Cardiology/American Heart Association Task Force on Practice Guidelines (Committee on the Management of Patients With Unstable Angina). Circulation. 2002;106:1893-900.]<br>● Unstable angina was diagnosed in the presence of symptoms and signs of acute cardiac ischemia and/or coronary disease, i.e. suggestive history and clinical examination, typical ischemic ECG changes at rest or during exercise, regional wall motion abnormality following stress echocardiography, or significant stenosis (>70%) on coronary angiography, without evidence of myocardial necrosis.<br>● Non-ST elevation myocardial infarction was diagnosed in the presence of signs and symptoms of acute cardiac ischemia and an elevated level of troponin on serial testing.
Sinha	2004	Total	208	● We recruited, on a prospective sequential basis, patients who arrived at the ED within three hours of acute chest pain.
		NICP	77	● Patients were classified as nonischaemic chest pain (NICP) when<br>(1) a reported noncardiac mechanism was confirmed as the cause of chest pain;<br>(2) all of the following criteria were met: negative cTnT results on serial sampling (over a 6–9 hour interval), presence of normal ECGs, and absence of any current (lesions <70% diameter reduction in any major epicardial vessel) or previous evidence of CAD;<br>(3) a diagnosis of ACS was objectively excluded after admission to the coronary care unit (CCU).
		ACS	131	● Practice guidelines for the redefinition of AMI (ESC/ACC) and the management of patients with UA (ACC/AHA), were used to diagnose ACS.<br>[The Joint European Society of Cardiology and American College of Cardiology Committee Myocardial Infarction. Redefined—a consensus document of the Joint European Society of Cardiology/American College of Cardiology Committee for the redefinition of myocardial infarction. J Am Coll Cardiol. 2000;36:959–69.]<br>[Braunwald E, et al. ACC/AHA guidelines for the management of patients with unstable angina and non-ST segment elevation myocardial infarction. J Am Coll Cardiol. 2000;36:970–1062.]<br>● STEMI was diagnosed if there was ST segment elevation >0.1 mV in two or more contiguous leads.<br>● NSTEMI was diagnosed if ECG was non-diagnostic and cTnT >0.05 ng/ml.<br>● UA was diagnosed in the presence of signs and symptoms of acute cardiac ischaemia without evidence of myocardial necrosis. Positive indications for UA were a suggestive history and clinical examination; typical ischaemic ECG changes at rest or during exercise; regional wall motion abnormality after stress

				echocardiography, abnormal perfusion scans or significant stenosis (>70%) on coronary angiography.
Sokhanvar	2012	Total	226	● We engaged, on a prospective basis, patients who arrived at the ER within three hours of acute chest pain.<br>● The excluded patients from our study were all of pregnant women, acute renal failure, patients with diagnosis of acute mesenteric ischemia, peripheral vascular disease, or any brain ischemia.
		NICP	106	● On the other hand patients were assessed as NICP when (1) a result of non cardiac mechanism was confirmed as the cause of chest pain;<br>(2) all of these criteria were acquired: negative cTnT findings on serial sampling (over a 6–9 hour interval), presence of normal ECGs, and absence of any recent changes (lesions < 50 % diameter reduction in any major epicardial vessel) or previous evidence of coronary artery disease;<br>(3) a diagnosis of ACS was objectively ruled out after admission to the coronary cardiac unit (CCU).
		ACS	120	● However in our study according to the Heas Chan and Braunwald references we introduced patients as UA with at least one of three features of chest pain:<br>(1) Occurring at rest (or minimal exertion) and usually lasting > 20 minutes (if not interrupted by Nitroglycerin administration);<br>(2) being severe and of new onset (within 1 month) and<br>(3) occurring with a crescendo pattern (more severe, prolonged, or frequent than previously).
Takhshid	2010	Total	123	● A total of 123 patients who arrived at the ED within 3 h of clinical signs suggestive of ACS were enrolled in this prospective double blind study.
		NICP	53	-
		ACS	70	● Diagnosis of ACS was made by two independent physicians blinded to the results of markers.<br>● Diagnosis of ACS was made with reference to the ACC/AHA 2007 guidelines for the diagnosis of unstable angina and acute myocardial ischemia and ESC/ACC 2007 guidelines for the redefinition of AMI.<br>[Anderson JL, et al. ACC/AHA 2007 guidelines for the management of patients with unstable angina/non-ST-Elevation myocardial infarction: a report of the American College of Cardiology/American Heart Association Task Force on Practice Guidelines (Writing Committee to Revise the 2002 Guidelines for the Management of Patients With Unstable Angina/Non-ST-Elevation Myocardial Infarction) developed in collaboration with the American College of Emergency Physicians, the Society for Cardiovascular Angiography and Interventions, and the Society of Thoracic Surgeons endorsed by the American Association of Cardiovascular and Pulmonary Rehabilitation and the Society for Academic Emergency Medicine. J Am Coll Cardiol. 2007;50:e1-157.]<br>[Thygesen K, et al. On behalf of the Joint ESC/ACCF/AHA/WHF Task Force for the redefinition of myocardial infarction. Eur Heart J. 2007;28:2525-38.]
Talwalkar	2008	Total	89	● In this retrospective study, we assessed 89 patients who came sequentially to the emergency room between December 2004 and January 2005. All these patients

				presented with chest pain and were subsequently admitted, either for observation in the emergency room or treatment in the hospital.
		NICP	66	• Patients in this group had chest pain with one or more of the following features: no classical symptoms of myocardial ischemia, non-diagnostic EKG, history of diabetes and/or hypertension, evidence of pleural, pericardial or pulmonary disease, and history or evidence of trauma.
		ACS	23	• This group included patients with stable, unstable, or variant angina, and/or acute myocardial infarction. These patients had chest pain with one or more of the following symptoms: radiation, chest pressure/tightness, shortness of breath, lower jaw pain, left arm pain, epigastric pain, syncope, hypotension, and palpitations.

Table S2. Comprehensive list presenting the search strategy.

Embase

No.	Searches	Results
1	Myocardial infarction.ti,ab.	257854
2	Heart infarction/ or acute heart infarction/ or infarction/ or ST segment elevation myocardial infarction/	376515
3	Myocardial disease/	5972
4	exp heart infarction/	369937
5	exp heart muscle ischemia/	91850
6	exp coronary artery disease/ or exp ischemic heart disease/	779184
7	exp angina pectoris/	95627
8	Angina.ti,ab.	71733
9	1 or 2 or 3 or 4 or 5 or 6 or 7 or 8	833801
10	IMA.ti,ab.	216
11	ischemia modified albumin.ti,ab.	859
12	ischaemia modified albumin.ti,ab.	118
13	cobalt binding.mp.	216
14	10 or 11 or 12 or 13	1208
15	9 and 14	390

Medline

No.	Searches	Results
1	Myocardial infarction.ti,ab	174744
2	*Infarction/	5697
3	Myocardial infarction/	163962
4	exp Acute Coronary Syndrome/ or exp Coronary Disease/ or exp Coronary Artery Disease/ or exp Myocardial Infarction/ or exp Myocardial Ischemia/	424306
5	exp Angina Pectoris/ or exp Angina, Unstable/ or Angina, Stable/	43160
6	Angina.ti,ab.	51867
7	1 or 2 or 3 or 4 or 5 or 6	486216
8	IMA.ti,ab.	127
9	ischemia modified albumin.ti,ab.	548
10	ischaemia modified albumin.ti,ab.	84
11	cobalt binding.mp.	139
12	8 or 9 or 10 or 11	798
13	7 and 12	220

Cochrane library

No.	Searches	Results
1	("myocardial infarction"):ti,ab,kw (Word variations have been searched)	29668
2	(angina):ti,ab,kw (Word variations have been searched)	13454
3	MeSH descriptor: [Infarction] explode all trees	3240
4	MeSH descriptor: [Ischemia] explode all trees	5126
5	MeSH descriptor: [Coronary Vessels] explode all trees	1453
6	MeSH descriptor: [Angina Pectoris] explode all trees	4522
7	(acute coronary syndrome):ti,ab,kw (Word variations have been searched)	7102
8	#1 or #2 or #3 or #4 or #5 or #6 or #7	44061
9	(ima):ti,ab,kw (Word variations have been searched)	797
10	(ischemia modified albumin):ti,ab,kw (Word variations have been searched)	78
11	("ischaemia modified albumin"):ti,ab,kw (Word variations have been searched)	57
12	(cobalt binding): ti,ab,kw (Word variations have been searched)	11
13	Mesh descriptor: [Serum Albumin, Human] explode all trees	83
14	#9 or #10 or #11 or #12 or #13	902
15	#8 and #14	71

Table S3. Characteristics of excluded from meta-analysis.

	Author	Year	Reason for exclusion
1	Abadie	2005	Irrelevant control group
2	Abdelhamid	2014	Data duplicated from the same studies
3	Ali	2014	Irrelevant control group
4	Aparci	2007	Irrelevant outcome measure
5	Aziz	2008	Irrelevant outcome measure
6	Bar Or	2000	Irrelevant population
7	Bayir	2015	Irrelevant control group
8	Behera	2012	Irrelevant control group
9	Bhagavan	2003	Irrelevant population
10	Cai	2010	Irrelevant outcome measure
11	Cevik	2013	Irrelevant outcome measure
12	Chawla	2006	Irrelevant outcome measure
13	Chen	2008	Irrelevant outcome measure
14	Chen	2009	Irrelevant outcome measure
15	Cho	2007	Irrelevant population
16	Cingozbay	2008	Irrelevant outcome measure
17	Da Silva	2011	Irrelevant population
18	Da Silva	2010	Irrelevant population
19	Dawie	2011	Conference abstracts
20	Demir	2018	Irrelevant population
21	El-sayed	2016	Irrelevant population
22	Fan	2014	Irrelevant outcome measure
23	Gencpinar	2012	Irrelevant outcome measure
24	Gholikhani	2018	Irrelevant control group
25	Goodacre	2013	Irrelevant outcome measure
26	Gorur	2016	Conference abstracts
27	Hausen	2012	Irrelevant outcome measure
28	Hazini	2015	Irrelevant population
29	He	2009	Irrelevant outcome measure
30	Huang	2009	Irrelevant outcome measure
31	Kalay	2007	Irrelevant population
32	Kazanis	2009	Irrelevant control group
33	Kim	2008	Irrelevant population
34	Kumar	2008	Irrelevant population
35	Kumar	2016	Irrelevant population
36	Lavall	2016	Irrelevant outcome measure
37	Liebetrau	2014	Irrelevant outcome measure
38	Lin	2010	Irrelevant outcome measure
39	Liu	2010	Conference abstracts
40	Liyan	2008	Irrelevant population
41	Magdy	2014	Conference abstracts
42	Maneewong	2011	Irrelevant control group
43	Mastella	2009	Irrelevant control group
44	Mishra	2018	Irrelevant population
45	Mojibi	2018	Irrelevant control group
46	Mowafy	2013	Irrelevant population
47	Panjwani	2013	Irrelevant outcome measure
48	Pankert	2011	Irrelevant outcome measure
49	Pantazopoulos	2009	Irrelevant outcome measure
50	Patil	2013	Irrelevant population
51	Peacock	2006	Review

52	Piechota	2006	Irrelevant population
53	Sharma	2007	Irrelevant population
54	Shen	2011	Irrelevant population
55	Shen	2007	Irrelevant population
56	Stozakovic	2013	Irrelevant outcome measure
57	Sygitowicz	2011	Conference abstracts
58	Takhshid	2011	Conference abstracts
59	Toker	2013	Irrelevant control group
60	Toklu	2010	Irrelevant population
61	Tripathy	2013	Irrelevant outcome measure
62	Van Belle	2010	Irrelevant population
63	Wudkowska	2010	Irrelevant population
64	Yan	2013	Irrelevant outcome measure
65	Yu	2011	Irrelevant outcome measure
66	Zhiqin	2010	Irrelevant outcome measure
67	Zhong	2012	Irrelevant population
1	Abadie	2005	Irrelevant control group

Table S4. Number of true positives, true negatives, false positives, and false negatives based on the ischemia-modified albumin cut-point for studies providing this data.

Study	Year	Target condition	IMA cut-off (U/ml)	TP (n)	FP (n)	FN (n)	TN (n)	Sen (%)	Spe (%)	PPV (%)	NPV (%)
Anwaruddin	2005	ACS	90	20	116	5	52	80.0	31.0	14.7	91.2
Bhakthavatsala	2014	ACS	80	57	3	8	21	87.7	87.5	95.0	72.4
Bhardwaj	2011	ACS	85	57	235	5	21	91.9	8.2	19.5	80.8
Chapentier	2010	ACS	85	128	315	57	177	69.2	36.0	28.9	75.6
Christenson	2001	AMI	75	29	58	6	131	82.9	69.3	33.3	95.6
Collinson	2006	AMI	85	18	324	19	177	48.6	35.3	5.3	90.3
Hjortshoj	2010	AMI	88.2	30	37	5	35	85.7	48.6	44.8	87.5
Keating	2006	ACS	86	35	203	7	32	83.3	13.6	14.7	82.1
Kim	2010	ACS	98.5	106	89	99	73	51.7	45.1	54.4	42.4
Kountana	2013	ACS	31.95	2	20	3	8	40.0	28.6	9.1	72.7
Lee	2007	ACS	85	120	183	9	101	93.0	35.6	39.6	91.8
Liyan	2009	ACS	70.5	73	5	9	21	89.0	80.8	93.6	70.0
Roy	2004	ACS	93.5	48	17	16	50	75.0	74.6	73.8	75.8
Sinha	2004	ACS	85	107	42	24	35	81.7	45.5	71.8	59.3
Sokhanvar	2012	ACS	85	65	14	55	92	54.2	86.8	82.3	62.6
Takhshid	2010	ACS	82.4	59	8	11	45	84.3	84.9	88.1	80.4
Talwalkar	2008	ACS	117	12	14	11	52	52.2	78.8	46.2	82.5

IMA, ischemia-modified albumin; TP, true positive; FP, false positive; FN, false negative; TN, true negative; NPV, negative predictive value; PPV, positive predictive value.

Figure S1. Assessment of study quality.

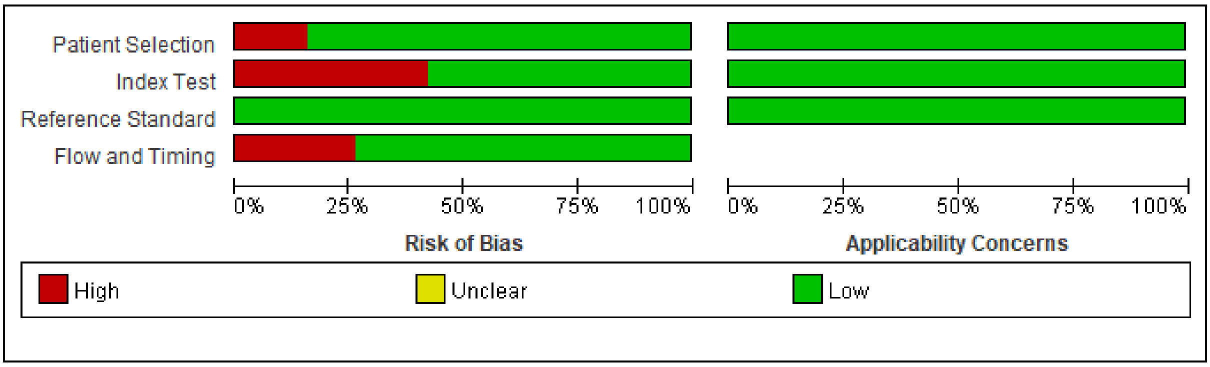

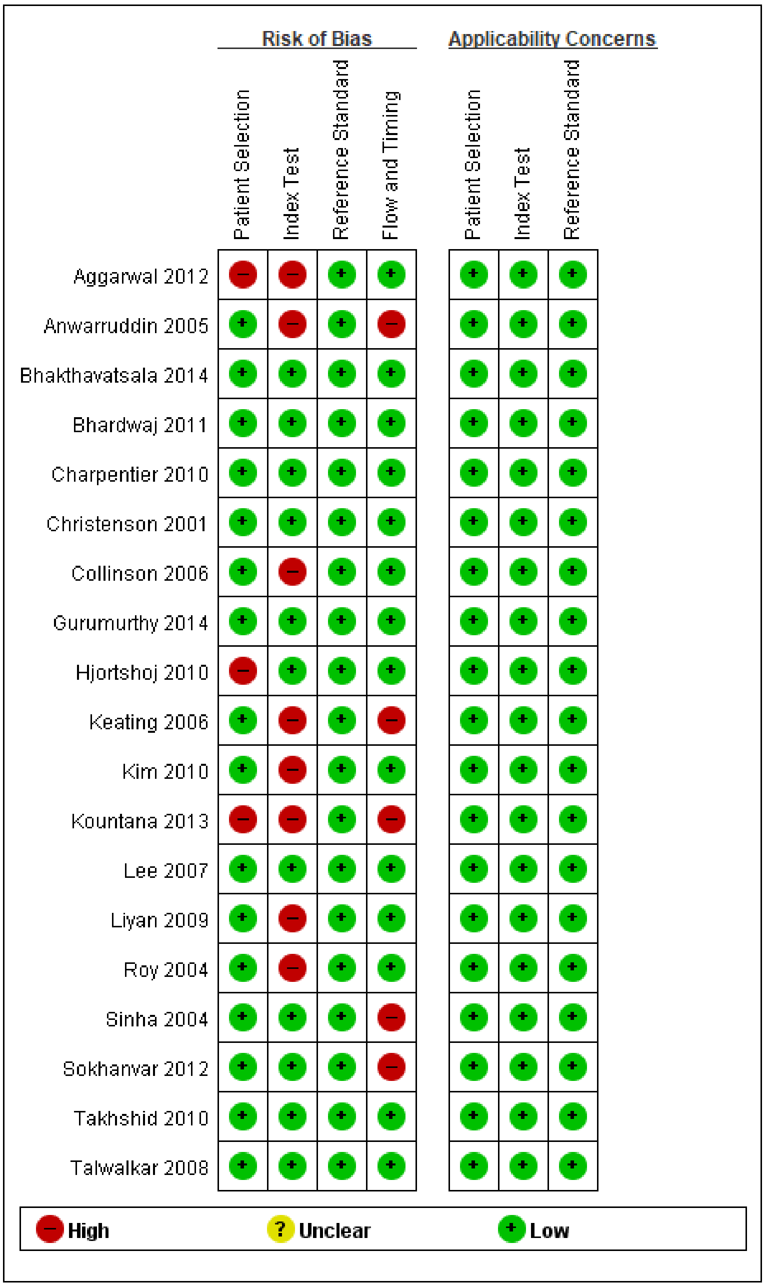

Figure S2. Forest plot of the association of serum IMA levels between ACS patients and NICP.

Study or Subgroup	ACS Mean	ACS SD	ACS Total	NICP Mean	NICP SD	NICP Total	Weight	Std. Mean Difference IV, Random, 95% CI
**2.1.1 Asia**								
Aggarwal 2012	30.87	3.48	50	4.99	2.07	50	7.1%	8.97 [7.64, 10.30]
Bhakthavatsala 2014	88.0769	8.9172	65	56.38	23.89	24	9.0%	2.17 [1.60, 2.74]
Gurumurthy 2014	89.4367	8.0978	405	77.94	6.69	135	9.5%	1.48 [1.26, 1.69]
Kim 2010	100.277	3.7389	181	99.47	2.24	162	9.5%	0.26 [0.05, 0.47]
Liyan 2009	64	4.53	82	74.67	5.49	26	9.0%	-2.22 [-2.75, -1.68]
Takhshid 2010	112.1	24.5	70	63.4	15.1	53	9.2%	2.31 [1.84, 2.77]
**Subtotal (95% CI)**			**853**			**450**	**53.2%**	**2.01 [0.68, 3.34]**
Heterogeneity: Tau² = 2.66; Chi² = 390.05, df = 5 (P < 0.00001); I² = 99%								
Test for overall effect: Z = 2.96 (P = 0.003)								
**2.1.2 Europe**								
Charpentier 2010	90.6	11	185	88.7	10.8	492	9.5%	0.17 [0.01, 0.34]
Hjortshoj 2010	90.6	45.22	35	86.36	21.35	72	9.2%	0.14 [-0.27, 0.54]
Roy 2004	98.3	11.4	64	85.5	15.2	67	9.3%	0.94 [0.58, 1.31]
Sinha 2004	96.1	3.97	131	87.03	4.76	77	9.3%	2.11 [1.76, 2.46]
**Subtotal (95% CI)**			**415**			**708**	**37.4%**	**0.84 [-0.06, 1.74]**
Heterogeneity: Tau² = 0.81; Chi² = 104.81, df = 3 (P < 0.00001); I² = 97%								
Test for overall effect: Z = 1.83 (P = 0.07)								
**2.1.3 North America**								
Bhardwaj 2011	95.67	12.14	62	99.33	11.93	256	9.4%	-0.31 [-0.58, -0.03]
**Subtotal (95% CI)**			**62**			**256**	**9.4%**	**-0.31 [-0.58, -0.03]**
Heterogeneity: Not applicable								
Test for overall effect: Z = 2.15 (P = 0.03)								
**Total (95% CI)**			**1330**			**1414**	**100.0%**	**1.30 [0.59, 2.00]**
Heterogeneity: Tau² = 1.36; Chi² = 560.84, df = 10 (P < 0.00001); I² = 98%								
Test for overall effect: Z = 3.59 (P = 0.0003)								
Test for subgroup differences: Chi² = 15.89, df = 2 (P = 0.0004), I² = 87.4%								

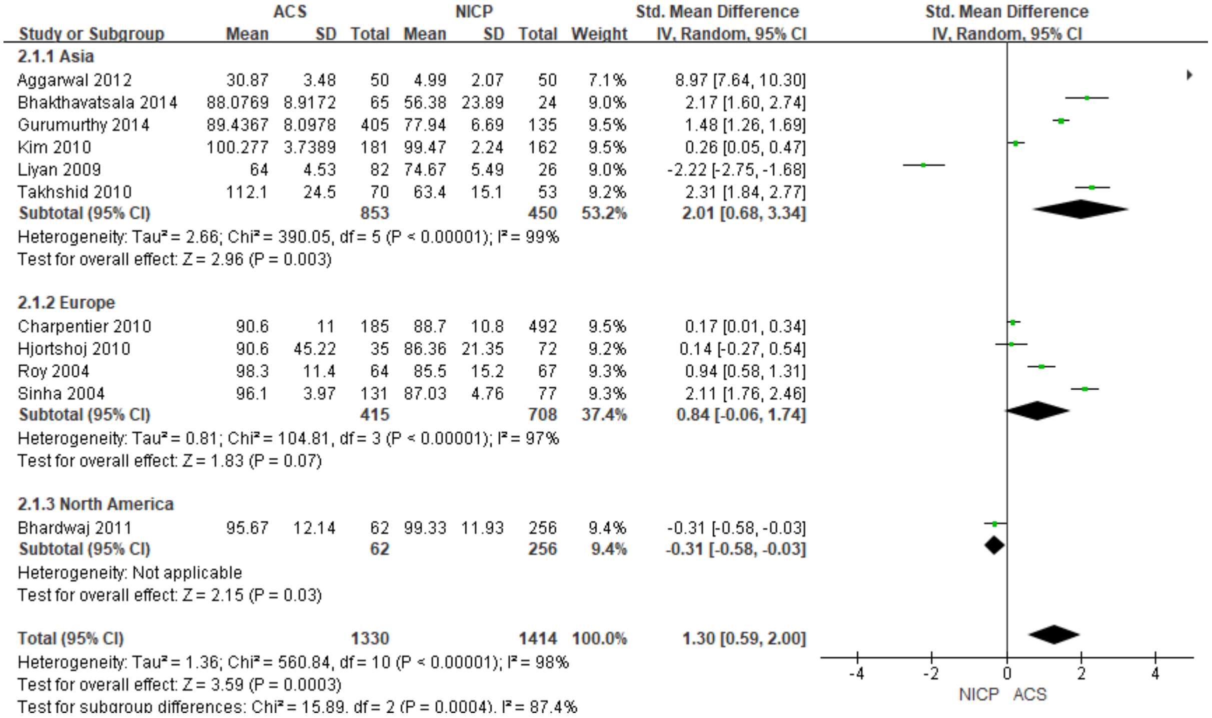

Figure S3. Forest plot of the association of serum IMA levels between ACS patients and NICP by three traditional types of ACS.

	ACS			NICP				Std. Mean Difference	Std. Mean Difference
**Study or Subgroup**	**Mean**	**SD**	**Total**	**Mean**	**SD**	**Total**	**Weight**	**IV, Random, 95% CI**	**IV, Random, 95% CI**
**3.1.1 UA vs NICP**									
Bhakthavatsala 2014	89	7.76	25	56.38	23.89	24	18.6%	1.82 [1.15, 2.50]	
Bhardwaj 2011	95	13.84	40	99.33	11.93	256	20.3%	-0.35 [-0.69, -0.02]	
Gurumurthy 2014	88.9	6.16	135	77.94	6.69	135	20.5%	1.70 [1.42, 1.98]	
Kim 2010	101.53	3.31	97	99.47	2.24	162	20.5%	0.76 [0.50, 1.02]	
Sinha 2004	97.37	4.83	85	87.03	4.76	77	20.1%	2.15 [1.76, 2.53]	
**Subtotal (95% CI)**			**382**			**654**	**100.0%**	**1.20 [0.33, 2.08]**	
Heterogeneity: Tau² = 0.95; Chi² = 128.73, df = 4 (P < 0.00001); I² = 97%									
Test for overall effect: Z = 2.70 (P = 0.007)									
**3.1.2 NSTEMI vs NICP**									
Bhardwaj 2011	95.67	11.09	22	99.33	11.93	256	24.7%	-0.31 [-0.74, 0.13]	
Gurumurthy 2014	87.31	5.95	135	77.94	6.69	135	25.8%	1.48 [1.21, 1.75]	
Hjortshoj 2010	90.6	45.22	35	86.36	21.35	72	25.0%	0.14 [-0.27, 0.54]	
Sinha 2004	93.1	13.73	26	87.03	4.76	77	24.6%	0.75 [0.30, 1.21]	
**Subtotal (95% CI)**			**218**			**540**	**100.0%**	**0.52 [-0.33, 1.37]**	
Heterogeneity: Tau² = 0.71; Chi² = 59.27, df = 3 (P < 0.00001); I² = 95%									
Test for overall effect: Z = 1.21 (P = 0.23)									
**3.1.3 STEMI vs NICP**									
Gurumurthy 2014	92.1	10.6	135	77.94	6.69	135	51.8%	1.59 [1.32, 1.87]	
Sinha 2004	90.93	15.88	20	87.03	4.76	77	48.2%	0.47 [-0.03, 0.96]	
**Subtotal (95% CI)**			**155**			**212**	**100.0%**	**1.05 [-0.05, 2.15]**	
Heterogeneity: Tau² = 0.59; Chi² = 15.14, df = 1 (P = 0.0001); I² = 93%									
Test for overall effect: Z = 1.87 (P = 0.06)									

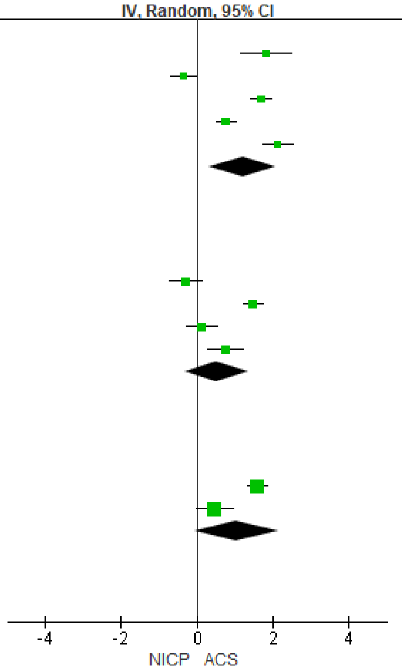

Figure S4. Random-effects univariate meta-regression between serum IMA values and (a) number of patients, (b) prevalence of ACS.

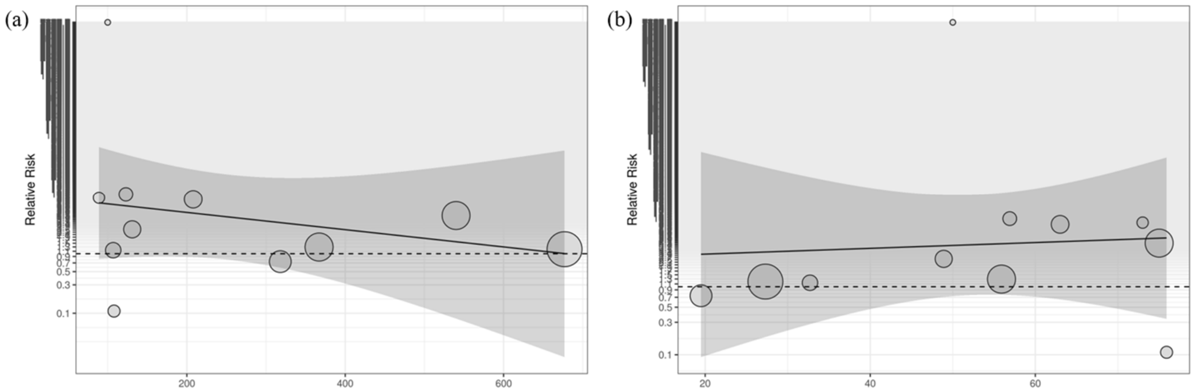

Figure S5. Forest plot of the association of serum IMA levels between ACS patients and NICP, with the exception of one study (Liyan et al.).

(a) The pooled DOR value of the serum IMA levels for diagnosis of ACS was 3.27 (I^2 = 90.6).

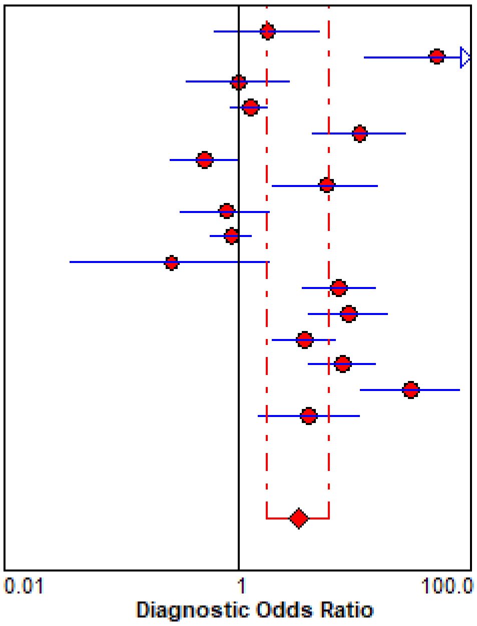

Diagnostic OR (95% CI)

1.79 (0.64 - 5.04)
49.88 (12.08 - 205.95)
1.02 (0.37 - 2.82)
1.26 (0.88 - 1.81)
10.92 (4.30 - 27.72)
0.52 (0.26 - 1.01)
5.68 (1.98 - 16.28)
0.79 (0.32 - 1.93)
0.88 (0.58 - 1.33)
0.27 (0.04 - 1.91)
7.36 (3.58 - 15.11)
8.82 (4.01 - 19.43)
3.72 (1.98 - 6.98)
7.77 (3.99 - 15.13)
30.17 (11.21 - 81.19)
4.05 (1.48 - 11.11)

Random Effects Model
Pooled Diagnostic Odds Ratio = 3.27 (1.77 to 6.03)
Cochran-Q = 159.11; df = 15 (p = 0.0000)
Inconsistency (I-square) = 90.6 %
Tau-squared = 1.3339

(b) Forest plot of the association of serum IMA levels between ACS patients and NICP.

	ACS			NICP				Std. Mean Difference
**Study or Subgroup**	**Mean**	**SD**	**Total**	**Mean**	**SD**	**Total**	**Weight**	**IV, Random, 95% CI**
**2.1.1 Asia**								
Aggarwal 2012	30.87	3.48	50	4.99	2.07	50	7.5%	8.97 [7.64, 10.30]
Bhakthavatsala 2014	88.0769	8.9172	65	56.38	23.89	24	9.8%	2.17 [1.60, 2.74]
Gurumurthy 2014	89.4367	8.0978	405	77.94	6.69	135	10.5%	1.48 [1.26, 1.69]
Kim 2010	100.277	3.7389	181	99.47	2.24	162	10.5%	0.26 [0.05, 0.47]
Takhshid 2010	112.1	24.5	70	63.4	15.1	53	10.1%	2.31 [1.84, 2.77]
**Subtotal (95% CI)**			**771**			**424**	**48.4%**	**2.81 [1.57, 4.05]**
Heterogeneity: Tau² = 1.90; Chi² = 246.15, df = 4 (P < 0.00001); I² = 98%								
Test for overall effect: Z = 4.43 (P < 0.00001)								
**2.1.2 Europe**								
Charpentier 2010	90.6	11	185	88.7	10.8	492	10.5%	0.17 [0.01, 0.34]
Hjortshoj 2010	90.6	45.22	35	86.36	21.35	72	10.2%	0.14 [-0.27, 0.54]
Roy 2004	98.3	11.4	64	85.5	15.2	67	10.3%	0.94 [0.58, 1.31]
Sinha 2004	96.1	3.97	131	87.03	4.76	77	10.3%	2.11 [1.76, 2.46]
**Subtotal (95% CI)**			**415**			**708**	**41.2%**	**0.84 [-0.06, 1.74]**
Heterogeneity: Tau² = 0.81; Chi² = 104.81, df = 3 (P < 0.00001); I² = 97%								
Test for overall effect: Z = 1.83 (P = 0.07)								
**2.1.3 North America**								
Bhardwaj 2011	95.67	12.14	62	99.33	11.93	256	10.4%	-0.31 [-0.58, -0.03]
**Subtotal (95% CI)**			**62**			**256**	**10.4%**	**-0.31 [-0.58, -0.03]**
Heterogeneity: Not applicable								
Test for overall effect: Z = 2.15 (P = 0.03)								
**Total (95% CI)**			**1248**			**1388**	**100.0%**	**1.62 [0.94, 2.29]**
Heterogeneity: Tau² = 1.13; Chi² = 447.64, df = 9 (P < 0.00001); I² = 98%								
Test for overall effect: Z = 4.68 (P < 0.00001)								
Test for subgroup differences: Chi² = 27.28, df = 2 (P < 0.00001), I² = 92.7%								

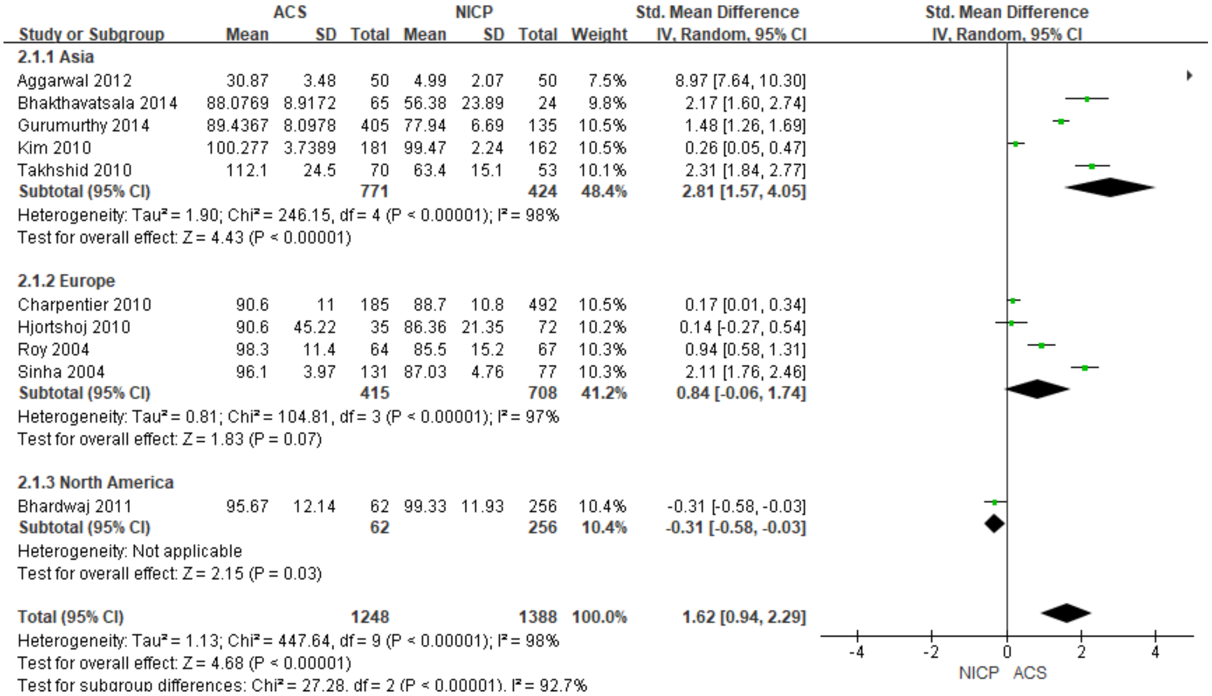

(c) Sensitivity analyses

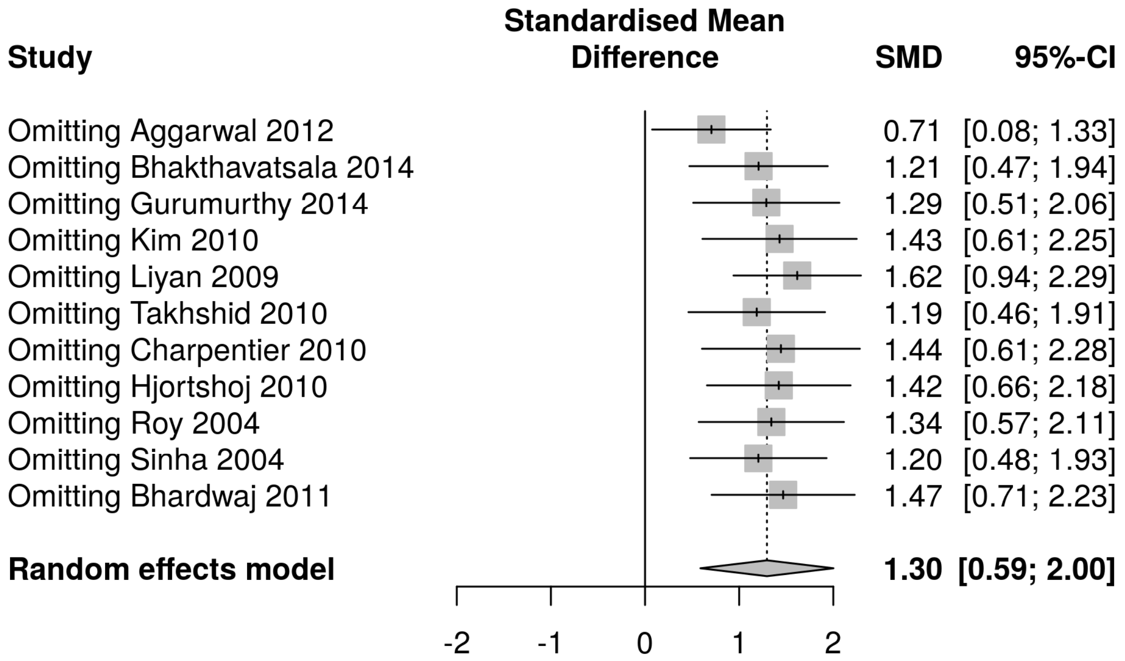

Supplement: Supplementary file 1 [file medicina-58-00614-s001.zip › medicina-1657873-supplementary.pdf]
